# Paraplegia From a Spinal Epidural Abscess Caused by Pasteurella multocida

**DOI:** 10.7759/cureus.15477

**Published:** 2021-06-06

**Authors:** Yechiel S Mor, Aliza Rizwan, Allan Frank

**Affiliations:** 1 Department of Internal Medicine, Detroit Medical Center/Wayne State University, Detroit, USA

**Keywords:** gram-negative bacteremia, pasteurella multocida, spinal abscess, acute paraplegia, pet, dog, haemophilus influenzae, intracranial epidural abscess, lower back pain (lbp)

## Abstract

*Pasteurella multocida* is a common cause of infection following bites or scratches caused by cats and dogs. It is a rarely reported and often overlooked pathogen. Typical presentation is a rapidly developing cellulitis at the infection site. Here we present a rare case of worsening lower extremity paraplegia due to a spinal epidural abscess caused by *P. multocida*. The patient was a 56-year-old female who had been experiencing several days of back pain, became septic and went on to develop paraplegia. Failure to improve prompted re-evaluation of the diagnosis with subsequent imaging notable for a spinal epidural abscess. Blood cultures grew *P. multocida* but were initially misidentified as *Haemophilus influenzae* and only with targeted antibiotic therapy and neurosurgical intervention did she begin to improve. Obtaining an animal history and knowing when to re-evaluate a diagnosis are essential skills for any clinician.

## Introduction

The literature contains few spinal epidural abscess cases and only one caused by *Pasteurella multocida*. Koji Oh et al reported the first case of a spinal epidural abscess due to *P. multocida* in a 68-year-old immunocompetent woman presenting with fever and sudden onset of severe back pain mimicking aortic dissection. Rapid initiation of antimicrobial therapy led to successful treatment without surgery [1].

We report an epidural abscess case caused by *P. multocida* which was first misidentified as *Haemophilus influenzae* leading to worsening sepsis despite treatment. The resultant delayed identification of a spinal epidural abscess required surgery, a change in antibiotic coverage, and a protracted hospital course. This first case of *P. multocida *causing paraplegia further highlights the importance of having spinal epidural abscess on the differential for back pain.

## Case presentation

A 56-year-old immunocompetent woman with fibromyalgia and history of left sciatica pain presented to the emergency department complaining of acute on chronic back pain. This pain had worsened over the past week and was now associated with progressive bilateral lower extremity weakness, confusion and urinary incontinence. She endorsed fevers, chills, cough, sputum production as well as burning on urination but denied any gastrointestinal symptoms or IV drug use. On presentation, she had a temperature of 36.7 degree C, a heart rate of 100 beats per minute, blood pressure of 98/66 mmHg, respiratory rate of 18 breaths per minute, and oxygen saturation of 97% on 2 liters nasal cannula oxygen. She was awake, cooperative but slightly somnolent, with delayed but appropriate verbal responses, Glasgow Coma Scale (GCS) 14. Pupils were equal round and reactive to light and there was no facial asymmetry or cranial nerve deficits. Auscultation did not reveal any murmurs. Motor strength in her lower extremities was 2/5 bilaterally. She did not have any other focal neurological deficits. All her pain appeared to originate from her thoracic spine and she would not allow us to check her reflexes citing her pain. There were no Osler’s nodes, splinter hemorrhages, Janeway lesions or Roth spots. She had no spinal tenderness, normal rectal tone but was laying on soiled bedsheets. She became septic with blood pressure of 84/56 mmHg, heart rate of 112 beats per minute, 96% oxygen saturation on room air, WBC 16K/CUMM, lactic acid 5.7gm/dl and was admitted to the ICU with severe sepsis of unknown source.

She was empirically started on broad-spectrum vancomycin and cefepime and deescalated to ceftriaxone 2 g daily when *H. influenza* resulted on blood cultures. Urine analysis was normal. EKG was normal sinus rhythm, with no ischemic changes. Chest X-ray showed mild pulmonary congestion. Infectious Disease was consulted and recommended continuing the ceftriaxone. The working diagnosis was a community-acquired pneumonia complicated by *H. influenzae* bacteremia. Her lactate improved with IV fluids, but her WBC increased from 13K/CUMM to 25K/CUMM. The worsened acute metabolic encephalopathy was initially attributed to sepsis and Hydromorphone, which had been given for pain when she was transferred to the medical floor.

The persistence of her back pain, leg weakness and fevers raised suspicion for an alternative source and the possibility of an abscess as a source of continued sepsis.

**Figure 1:**
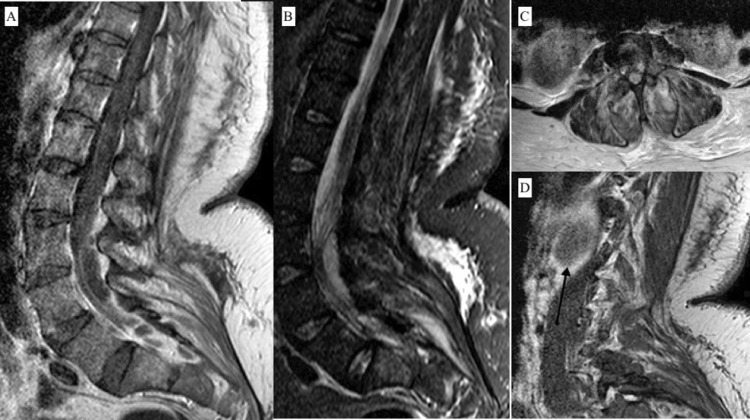
Her MRI showing a 10 x 1.3 cm dorsal epidural abscess between L2 and the distal-most thecal sac causing severe compression of the thecal sac. A. T1 image with low signal collection impinging on the thecal sac. B. T2 image with high signal collection impinging on the thecal sac. C. Collection seen posterior to the cord. D. Sagittal image of the spinal abscess.

A CT head was performed to investigate her worsening somnolence, which showed no acute intracranial process. Due to bacteremia, worsening weakness, and severe back pain a thoracic and lumbar MRI was ordered which showed a complicated spinal epidural abscess with cord compression: the dorsal epidural abscess spanned approximately 10 cm between mid-L3 level and the distal-most thecal sac, measuring approximately 1.2 cm in the maximum axial diameter and causing severe compression of the thecal sac. A bone marrow signal abnormality with enhancement in the S1 vertebral body, worrisome for early osteomyelitis. No associated discitis or bone destruction. A large soft tissue abscess in the distal right psoas muscle, measuring up to 5.4 cm in the maximum axial dimension. Mild diffuse edema of the left psoas muscle at the same level. Diffuse inflammatory/ infectious changes in the paraspinal muscles caudal to L3 level bilaterally containing multiple clusters of small abscesses on both sides. Mild widening of bilateral L4-5 and L5-S1 facet joints containing small amounts of fluid and mild peripheral enhancement, worrisome for septic arthritis (Figure 1).

She was transferred to the neurosurgical ICU for further care, neurosurgery was consulted and she was started on hourly neuro checks.

During this time her blood cultures were finalized as *P. multocida* and had initially been misidentified as *H. influenza*. Targeted antibiotic therapy with ampicillin-sulbactam was started.

Neurosurgery performed L3-L5 laminectomy and evacuation of the epidural abscess. Interventional radiology performed drainage of the left subscapular and right psoas abscesses. Abscess cultures were performed and confirmed *P. multocida* as the causative organism.

After evacuation of the epidural abscess, her paraplegia began improving and she was discharged to an inpatient rehab facility where over several months she gained full recovery.

## Discussion

*P. multocida* is a zoonotic agent of human disease and has been isolated from the upper respiratory tract and digestive system of domestic animals. It is estimated that there are some 70 million dogs and 74 million cats in the United States, and approximately 37% of all households in the United States have a dog, and 30% have a cat [2]. The most common consequence of *P. multocida* infection in humans is a local cellulitis, although serious systemic diseases can occur such as meningitis, empyema, pneumonia, peritonitis, osteoarticular infections, endocarditis, and septicemia.

In our case, we speculate the source of infection with *P. multocida* was her dog's saliva which may have found a portal of entry after licking a wound or unknown skin opening. Infections caused by *P. multocida* tend to spread quickly, disseminating through soft tissue and proximally along lymphatic vessels. *P. multocida* is known for causing soft tissue infections but an awareness of the more serious infections is paramount to appropriate treatment.

A retrospective study by Giordano et al 2015 showed that *P. multocida* infections not associated with an animal bite were often associated with bacteremia, severe comorbidities, immune-incompetent states, need for ICU management, and substantial mortality [3]. Asides from this study there are only a limited number of single case reports in the literature describing the clinical features and outcomes of patients presenting with systemic infection due to *P. multocida*; larger studies are lacking [2].

In our case, the initial culture was misidentified as *H. influenzae*. Both *H. influenzae* and *P. multocida* are gram-negative organisms. *P. multocida* produces a protein with homology to the P6 outer membrane protein of *H. influenzae* [4]. Only two case reports returned in a search of Pasteurella species being misidentified as Haemophilus. In our case, the presence of Haemophilus species misled a workup. The bacteremia and sepsis were attributed to pneumonia; only after the fevers and sepsis persisted did the search for an alternative diagnosis resume. This delay in diagnosis of the epidural abscess, and therefore delay in appropriate antibiotic management extended our patient’s medical course. Initially, the lower extremity weakness was mild and attributed to sepsis fatigue.

Most Haemophilus species, particularly *H. influenzae* require chocolate agar or some other source for X and V factors, while Pasteurella species do not [5]. However, a number of Haemophilus species will grow sufficiently on most blood agar media for growth to be discernible, thus necessitating further differentiation from Pasteurella by testing for X and V factor dependency. No conclusive diagnostic identification is possible through selective culturing, phenotyping, or direct microscopic examination alone [4].

When Pasteurella is isolated, susceptibility testing is generally not necessary given the typical susceptibility pattern of the organism. Treatment for local infections has typically been with penicillin. For more invasive infections such as septic arthritis or osteomyelitis, ampicillin-sulbactam, piperacillin-tazobactam, or broad-spectrum cephalosporins (such as ceftriaxone) are appropriate [5].

Our patient did not have meningitis but in such a scenario, third-generation cephalosporins with good central nervous system (CNS) penetration, such as ceftriaxone would be warranted for 21 days with repeated lumbar puncture to ensure improvement.

For early detection, it is paramount to include a spinal epidural abscess in the differential diagnosis for back pain. Our patient presented with sepsis and bacteremia which overshadowed her complaint of back pain and leg weakness and only after the leg weakness worsened to a paraplegia with ongoing septic signs was imaging pursued. Spinal epidural abscesses are widely reported in the literature. Most cases have one or more predisposing conditions, such as underlying diabetes mellitus, alcoholism, HIV or a recent intervention such as trauma, surgery, drug injection, catheter placement or a potential local or systemic source of infection. Bacteria gain access to the epidural space through contiguous spread in one-third of cases, hematogenous spread in half of the cases and the remaining cases never have a source of origination identified.

A diagnosis of spinal epidural abscess is suspected on the basis of clinical findings and supported by laboratory data and imaging results. Bacteremia is seen in more than 60% of spinal epidural abscess cases [3]. This case illustrates that if a patient does not improve despite appropriate therapy a reassessment of the diagnosis is warranted. The best clinical outcome depends on early intervention.

## Conclusions

We present an important and previously undescribed consequence of *P. multocida* infection resulting in a prolonged recovery period. Cases of Pasteurella causing an epidural abscess are few in the literature and here we have described a rare instance of* P. multocida* SEA progressing to a paraplegia. Obtaining the patient history of recent animal contact is essential in recognizing this possible source of infection.
